# HmtVar: a new resource for human mitochondrial variations and pathogenicity data

**DOI:** 10.1093/nar/gky1024

**Published:** 2018-10-29

**Authors:** Roberto Preste, Ornella Vitale, Rosanna Clima, Giuseppe Gasparre, Marcella Attimonelli

**Affiliations:** 1 Department of Biosciences, Biotechnology and Biopharmaceutics, University of Bari, Bari 70126, Italy; 2 Department of Medical and Surgical Sciences – DIMEC, Medical Genetics Unit, University of Bologna, Bologna 40126, Italy

## Abstract

Interest in human mitochondrial genetic data is constantly increasing among both clinicians and researchers, due to the involvement of mitochondrial DNA (mtDNA) in a number of physiological and pathological processes. Thanks to new sequencing technologies and modern databases, the large amount of information on mtDNA variability may be exploited to gain insights into the relationship between mtDNA variants, phenotypes and diseases. To facilitate this process, we have developed the HmtVar resource, a variant-focused database that allows the exploration of a dataset of over 40 000 human mitochondrial variants. Mitochondrial variation data, initially gathered from the HmtDB platform, are integrated with in-house pathogenicity assessments based on various evaluation criteria and with a set of additional annotations from third-party resources. The result is a comprehensive collection of information of crucial importance for human mitochondrial variation studies and investigation of common and rare diseases in which the mitochondrion may be involved. HmtVar is accessible at https://www.hmtvar.uniba.it and data may be retrieved using either a web interface through the Query page or a state-of-the-art API for programmatic access.

## INTRODUCTION

The mitochondrion, traditionally defined as the powerhouse of the eukaryotic cell, plays a role in a number of biological processes, and shows extensive variation in proteomic composition and function, differentiated in tissues and cell types. By virtue of such pivotal involvement, its dysfunction causes or contributes to human pathologies, such as neurodegenerative diseases, diabetes, cancer and metabolic syndromes. Hence, there is currently great interest in the relationship between mitochondria and disease, as confirmed by clinical literature (1–3).

Recent advances in high-throughput sequencing techniques have provided an unprecedented amount of genetic data capable of offering invaluable insights into different life science questions. This becomes even more significant in the light of the high number of sequences and related metadata available in public databases. Most of these data regard genomic variability, which can be exploited to achieve a better understanding of the correlations between DNA variants, phenotypes and diseases.

Big public biological datasets can be used to assess human diversity. A large number of mtDNA variations may simply represent neutral population specific polymorphisms, as reported by Phylotree (4), and can therefore be flagged as polymorphic variants, while the rest may be more or less involved in pathogenic processes. A huge amount of online resources for mitochondrial data analysis has surfaced over the years, with the common aim of producing a comprehensive knowledge of the onset and development of diseases in which mitochondria are involved. Some examples include the Mitomap (5) portal with the MitoTip (6) pathogenicity predictor for tRNA variants, the MSeqDR platform (7) and the HmtDB database (8); however, there is still no specific resource for specifically querying and retrieving mitochondrial variants annotated with dedicated functional, structural, population, disease-related information and classified according to well-assessed tiers of pathogenicity.

To this aim, we have designed and implemented the HmtVar resource (https://www.hmtvar.uniba.it), an online database that will guide clinicians and researchers in the assessment of variability and pathogenicity in human mitochondrial DNA.

## MATERIALS AND METHODS

### Data sources

HmtVar is a variant-centred database which offers mtDNA variability and pathogenicity data. The information available in HmtVar comes from several human mitochondrial genomes and exploits different third-party resources. HmtVar variants are gathered from variations found in completely sequenced human mitochondrial genomes, downloaded from GenBank (9) into HmtDB (8) (https://www.hmtdb.uniba.it) and annotated as either ‘healthy’, when derived from individuals with no description of disease, or ‘pathologic’ otherwise.

The HmtVar dataset is further enriched with non-synonymous and tRNA variants that are not yet observed among the HmtDB genomes, but can potentially occur, considering every possible substitution for each site of the reference human mitochondrial genome. With respect to ribosomal RNAs and regulatory regions loci, variants observed in HmtDB or reported outside HmtDB are annotated with the minimum number of attributes retrieved from external resources. The discrimination of observed from potential variants is performed using the frequency of the allele causing the variation, ranging from 0 to 1.

Any nucleotidic change is defined according to the revised Cambridge Reference Sequence (rCRS, Accession Number NC_012920.1) (10).

A set of attributes has been defined for each variant, based on evidence extracted from external resources or estimated by applying, on the complete human mitochondrial genomes of both ‘healthy’ and ‘pathologic’ individuals, computational approaches such as the estimation of nucleotide and amino-acidic sites variability. These values are estimated using, respectively, the SiteVar (11) and the MitVarProt (12) algorithms. The resulting variability scores (nt_var and aa_var) range from 0 to 1, with a higher value indicating a lower functional constraint of the site. Alongside with variability data, allele frequencies are also calculated and stored in HmtVar.

Table 1 reports the list of attributes distinguished according to the different functional loci: protein-coding (CDS), tRNAs, rRNAs and regulatory regions.

**Table 1.** List of attributes associated to HmtVar variants for each locus type

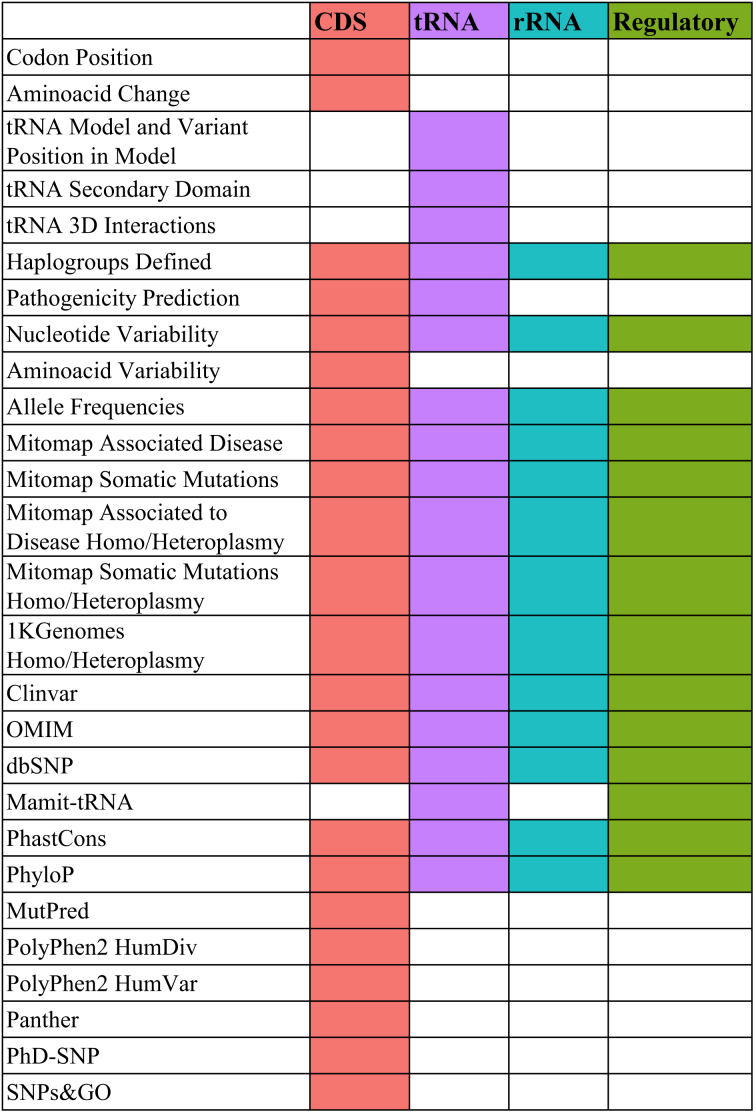

## VARIANT PATHOGENICITY ESTIMATION

### Disease score estimation

Disease Score Estimation (DSE) for non-synonymous variants is based on the algorithm implemented by Santorsola *et al.* (13), while DSE for mt-tRNA variants is based on criteria reported by Diroma *et al.* (14), derived from Yarham *et al.* (15) but normalized within the range 0–1 (Table 2A). mt-tRNA variants for which no functional evidence is available from literature are flagged as VUS (Variants of Undefined Significance).

**Table 2.** (A) tRNA scoring system implemented in HmtVar according to Yarham and Diroma criteria. The conservation criteria were modified thanks to the availability of Phastcons (31) and Phylop (32). (B) tRNA variant attributes to be considered for future improved disease score estimation

A	tRNA Scoring Criteria	Yes	No	Yes Normalized
	**tRNA structure parameters and reports**			
	Variant described as pathogenic by more than 1 report	2	0	0.1
	**Frequency and population data**			
	PhastCons conservation	1	0	0.05
	PhyloP conservation	1	0	0.05
	**Variant Heteroplasmy**			
	Heteroplasmy evidences	2	0	0.1
	**Evidences from functional studies**			
	Segregation of mutation with disease	2	0	0.1
	Histochemical evidence of mitochondrial disease	2	0	0.1
	Biochemical defect in OXPHOS complexes I, III or IV	2	0	0.1
	Pathogenicity evidence in trans mitochondrial cybrids or mutant mt-tRNA steady state level studies	5	0	0.25
	Evidence of mutation segregation with biochemical defect from single-fiber studies	3	0	0.15
**B**	**Additional tRNA variants Scoring Criteria**			
	**tRNA structure parameters and reports**			
	Cloverleaf-shaped secondary structure variation			
	Post-transcriptional modification			
	3D interaction involved in folding			
	**Frequency and population data**			
	Allele variant frequency > 2%			
	Allele variant frequency in patients > allele variant frequency in healthy individuals			
	Macrohaplogroup-defining variant			

### Disease score and allele frequency threshold

Once defined, the Disease Score (DS) values represent a tool to discriminate benign from pathogenic variants. The Disease Score Threshold (DS_T) was defined for this purpose. The DS_T value was set at 0.43 for non-synonymous variants, and at 0.35 for tRNA variants, according to protocols defined in (13) and (14), respectively. In order to reinforce the variant pathogenicity assignment, both protocols associate DS_T values with the empirical cumulative distribution of the nucleotide variability which in HmtVar is replaced by the allele frequency. Such procedure made it possible to determine, according to the latest HmtDB update, an Allele Frequency threshold (AF_T), estimated by considering only variants with DS greater than DS_T, equal to 0.003264 for non-synonymous variants and to 0.005020 for tRNA variants.

### Tier definition

In order to assign each non-synonymous or tRNA variant to a specific tier of pathogenicity, DS_T and AF_T were considered, according to the general rules listed in Table 3A.

**Table 3.** (A) General rules for variant pathogenicity assignment. (B) Non-synonymous and tRNA variant specific rules for pathogenicity tier assignment

A	Tier	Disease Score range	Allele Frequency range
	**General rules**		
	Polymorphic	DS < DS_T	AF > AF_T
	Likely Polymorphic	DS < DS_T	AF ≤ AF_T
	Likely Pathogenic	DS ≥ DS_T	AF > AF_T
	Pathogenic	DS ≥ DS_T	AF ≤ AF_T
**B**	**Non-Synonymous variants**		
	Polymorphic	DS < 0.43	AF > 0.003264
	Likely Polymorphic	DS < 0.43	AF ≤ 0.003264
	Likely Pathogenic	DS ≥ 0.43	AF > 0.003264
	Pathogenic	DS ≥ 0.43	AF ≤ 0.003264
	**tRNA variants**		
	Polymorphic	DS < 0.35	AF > 0.005020
	Likely Polymorphic	DS < 0.35	AF ≤ 0.005020
	Likely Pathogenic	DS ≥ 0.35	AF > 0.005020
	Pathogenic	DS ≥ 0.35	AF ≤ 0.005020

Using the specific thresholds defined, the final pathogenicity tiers were assessed as detailed in Table 3B.

Further criteria relevant for an exhaustive interpretation of mt-tRNA variants were considered, estimated and annotated, conditional to the availability of specific information. The additional criteria are listed in Table 2B.

### Implementation

HmtVar is built using the Python Flask framework (http://flask.pocoo.org/) and uses the SQLite (https://www.sqlite.org/index.html) database to manage data. The web interface was developed using Bootstrap (https://getbootstrap.com/) and the update procedure takes advantage of the Nextflow pipeline management system (https://www.nextflow.io/index.html).

The decision to implement HmtVar back-end functionality using Python Flask was made due to the need for a lightweight yet efficient framework both to build a reliable web service and to retrieve and analyse information from the underlying database. Python Flask integrates very well with SQLite databases and offers a set of tools to perform database construction, querying, editing and versioning right out of the box. SQLite, on the other hand, allows for a simpler and faster data storage and retrieval than legacy database engines; this is fundamental given the large amount of genomic information collected.

In addition, it was possible to deploy a comprehensive Application Programming Interface (API) without relying on other services, so that researchers and developers can access HmtVar data in a programmatic mode and integrate them into their applications.

HmtVar was developed with one of the key ideas being easy access from every device, so the Bootstrap library was employed for developing its front-end. Consequently, HmtVar can be accessed from either desktop or mobile devices without any loss in functionality.

One of the aims of HmtVar is to provide users with data that are constantly updated. This means collecting new variant entries and variability data as soon as they are available in HmtDB, as well as gathering additional information from several third-party resources. A specific software to retrieve new information from each data source was initially developed; this set of scripts was then aggregated using the Nextflow pipeline manager, which is capable of parallelizing the different retrieval and parsing tasks, speeding up the entire updating process considerably.

### Testing

HmtVar was tested on variants obtained by selecting all cases of oncocytic tumour (*N* = 117) from the HmtDB database, which account for ∼2.5% of all diseased subjects.

The 117 oncocytic tumours were of different types: 16 bilateral multifocal renal oncocytomas, 25 oncocytic pituitary adenomas, 4 parotid oncocytomas, 9 renal oncocytomas, 12 Warthin tumours, 1 rhino pharynx oncocytoma, 16 oncocytic hyperplastic thyroid nodules, 7 oncocytic follicular thyroid adenomas, 22 oncocytic thyroid carcinomas and 5 breast carcinomas with oncocytic characteristics.

## RESULTS AND DISCUSSION

### Variant statistics

**Figure 1.**
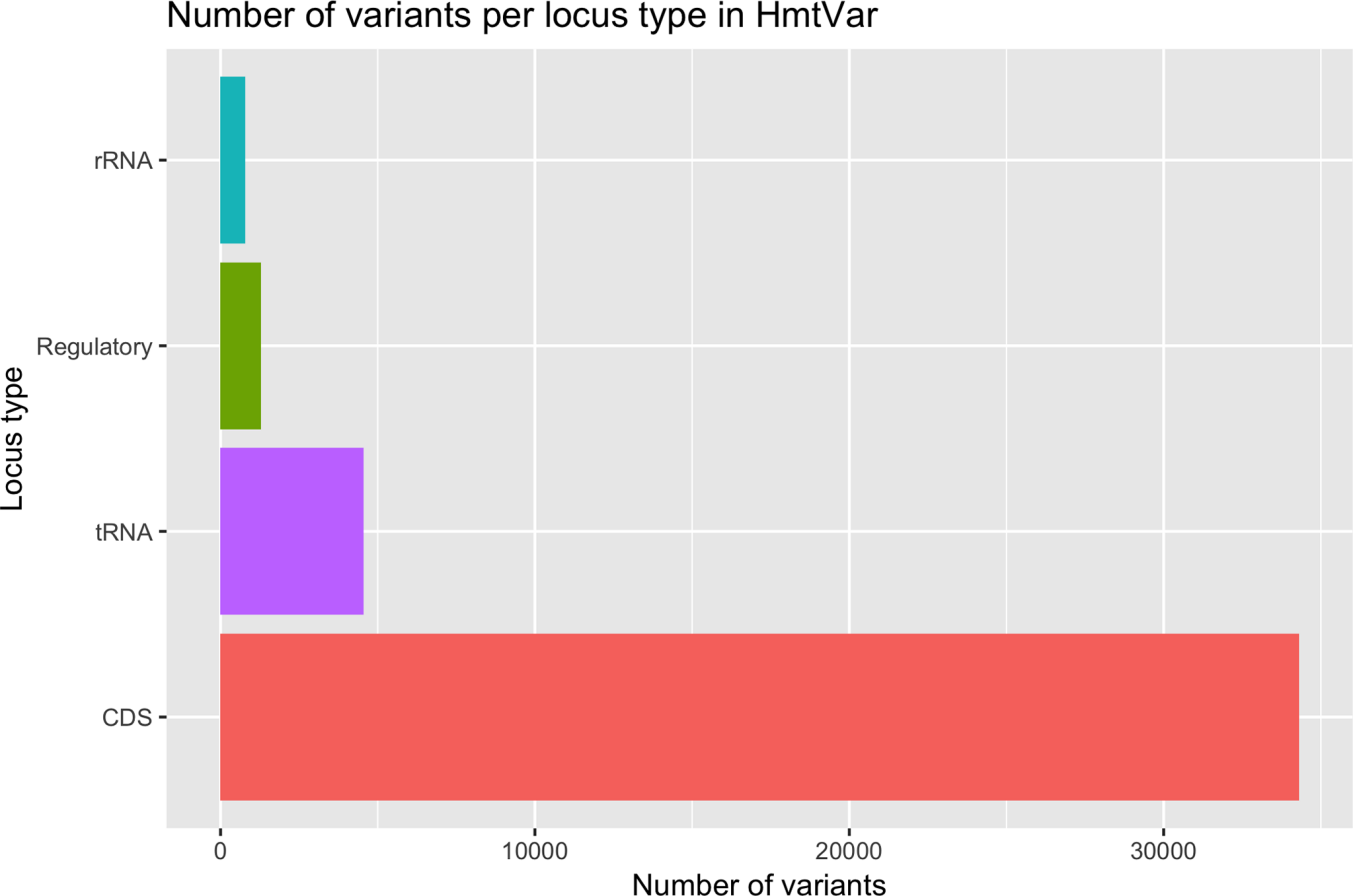
Total variants in HmtVar per locus type.

As of July 2018, HmtVar hosts a total of 40 923 mitochondrial variants distinguished as observed or potential variants. The observed variants are derived from 45 841 complete human mitochondrial genomes, of which 41 287 ‘healthy’ and 4554 ‘pathologic’. The total number of annotated variants per locus type is reported in Figure 1.

Based on the total length of rCRS (16 569 bp), the total number of potential variants, excluding insertions and deletions, is 49 707; HmtVar hosts annotations for 82.33% of them. The number of CDS and tRNA annotated variants matches the expected value based on rCRS gene lengths. The number of rRNA and regulatory regions variants annotated in HmtVar represent only a subset of the entire potential set due to the lack of specific information either in literature or from external resources. This information will be integrated with subsequent HmtVar updates as soon as new data will be published.

**Figure 2.**
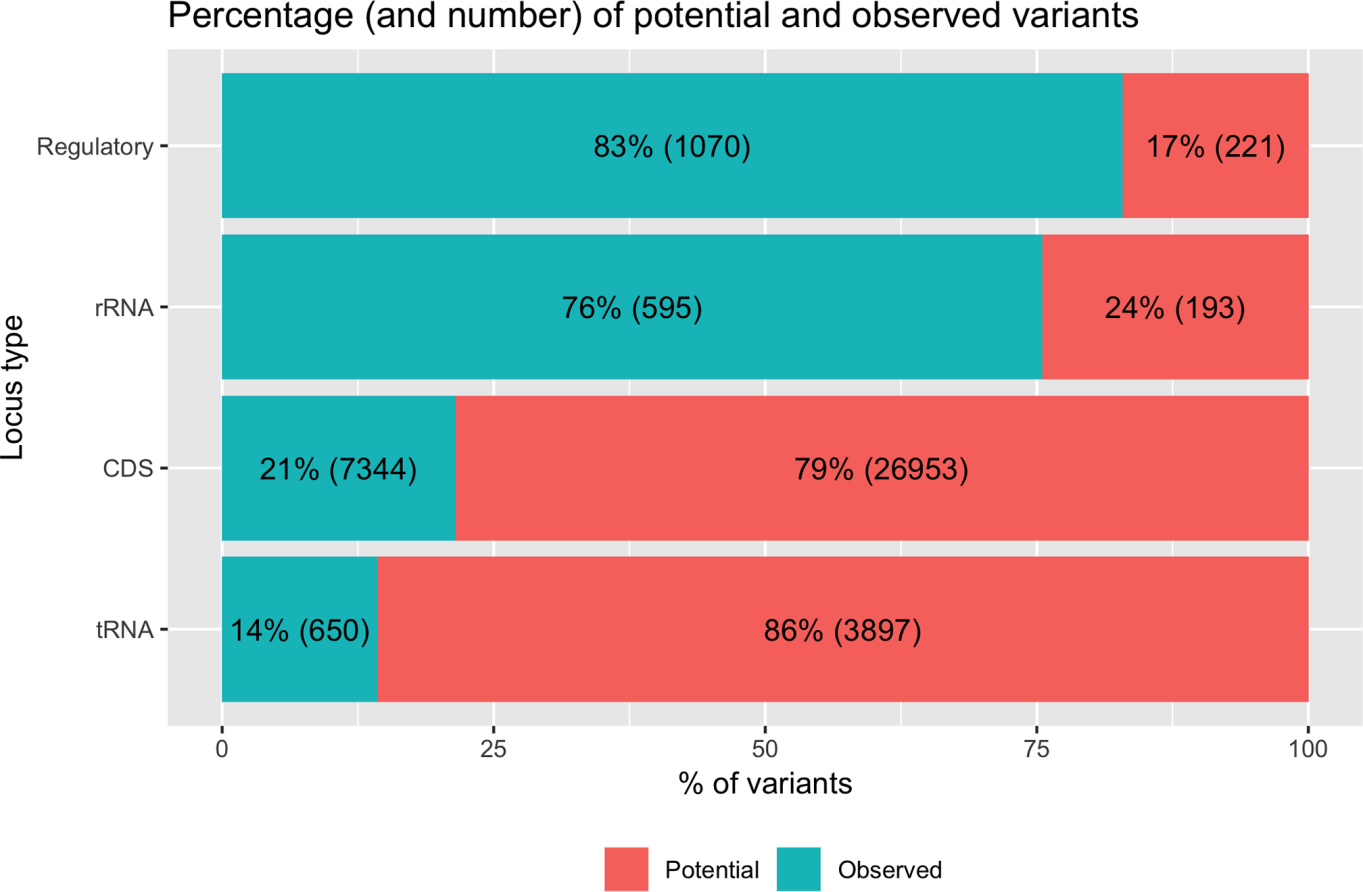
Percentage and number of potential and observed variants per locus type.

**Figure 3.**
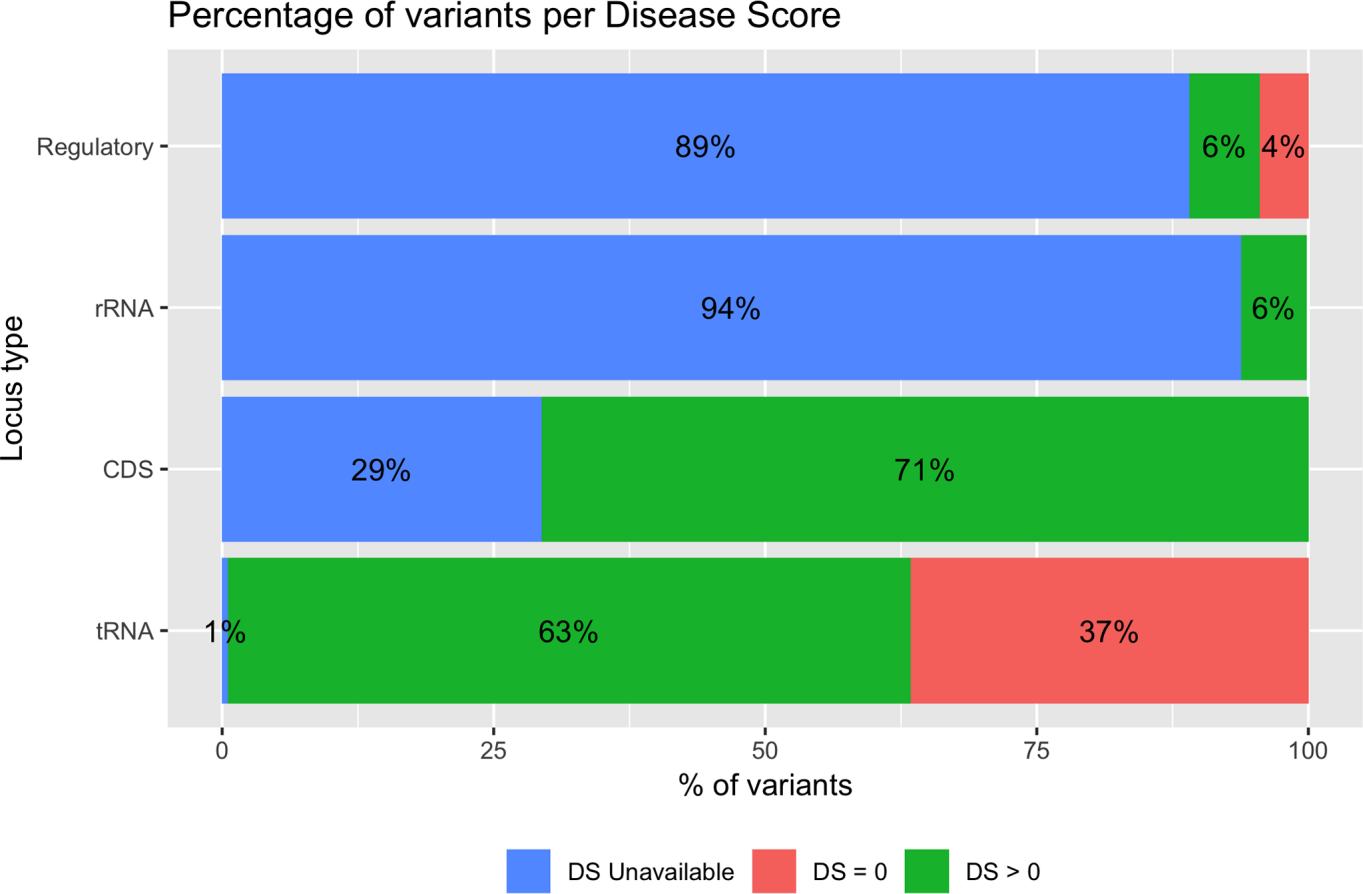
Percentage of variants with unassigned DS, with DS equal to 0 and with DS greater than 0, for each locus type.

Figure 2 shows the percentage of observed (AF > 0) and potential (AF = 0) variants as reported in HmtVar, while in Figure 3 it is possible to see, for each locus type, the percentage of variants annotated in HmtVar with unassigned DS, with DS equal to 0 and with DS greater than 0. Disease scores are not available for frameshift, stop-gain and stop-loss CDS variants.

Moreover, the unavailability of published functionals studies for a large number of tRNA variants did not allow to estimate a congruent disease score, whereby these variants are annotated as VUS despite DS > 0.

### Variant pathogenicity

**Figure 4.**
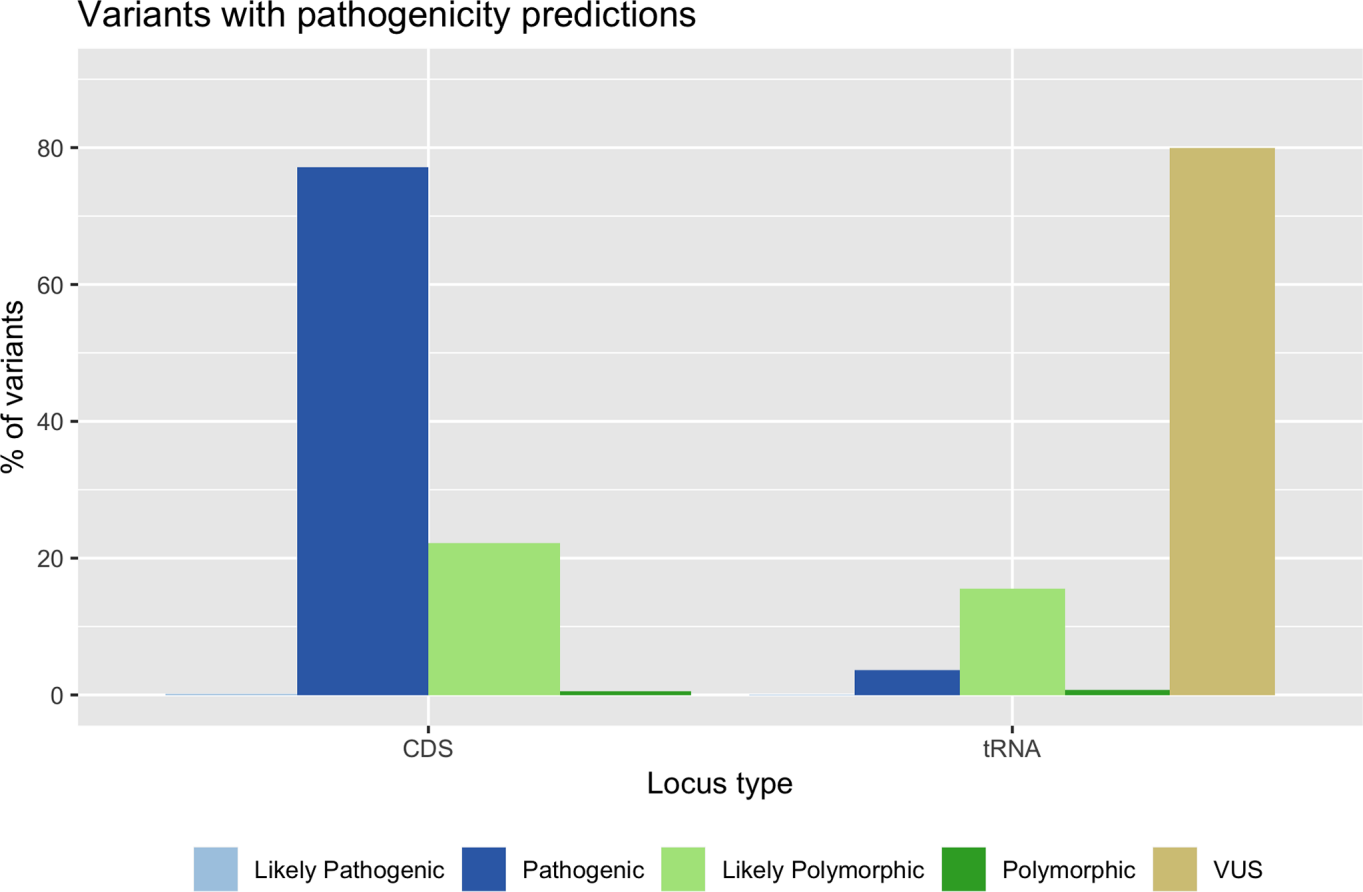
CDS and tRNA variants annotated in HmtVar with available pathogenicity predictions.

Tiers of pathogenicity based on criteria reported in Table 3 were estimated and annotated for 28 768 variants allowing their classification as pathogenic (18 857), likely pathogenic (30), polymorphic (164), likely polymorphic (6088), VUS (3629). Figure 4 shows the distribution of CDS and tRNA variants according to the five pathogenicity tiers.

For both tRNA and protein-coding genes, 92.8% of pathogenic variants are not observed in ‘healthy’ genomes, unlike the remaining 7.2%.

### Assessment of pathogenicity

The best way to test HmtVar was to select a dataset of mitochondrial genomes that would harbour pathogenic variants, to be flagged properly by our database. Based on our extensive experience in the field of somatic mutations in oncocytic tumours, a subset of human neoplasms with the unique ability to accumulate damaging mtDNA mutations within their highly deranged mitochondria, was selected HmtDB. The occurrence of disruptive mtDNA mutations, some of which very rare, as a genetic hallmark of oncocytomas, is widely recognised in literature, and supported by functional studies that reveal how such homoplasmic or near-homoplasmic changes are favoured exclusively in oncocytic tumours and not in other human cancers, and determine a dramatic respiratory dysfunction, often involving oxphos complexes disassembly (16–19). So, all genomes sequenced directly from the tumour tissue of oncocytoma patients (20–22), were retrieved from HmtDB and their variants were tested using HmtVar.

The genome analysis led to the identification of 313 substitutions in 106 patients (Supplementary Table S1). In the protein coding regions, 187 non-synonymous substitutions (59.7%), 22 nonsense (7%) and 34 frameshift mutations (10.9%) were identified using the MToolBox pipeline (23). Among all the non-synonymous variants found, HmtVar classified 72 of them as pathogenic or likely-pathogenic. As expected from the biology of oncocytic tumours, 32 out of 116 patients (28%) presented more than half of the non-synonymous mutations (40/72, 55%) localised within the subunits of complex I (Supplementary Table S1). The remaining non-synonymous pathogenic mutations mapped in genes encoding subunits of complex V (10 in *MT-ATP6* and 3 in *MT-ATP8*), of the Cytochrome c oxidase (4 in *MT-CO1* and 3 in *MT-CO2*) and of the only component of complex III (12 in *MT-CYB*) (Supplementary Table S1). Additionally, 52 patients harboured a substitution in tRNA genes. Among the 70 tRNA events, 61 were tagged as polymorphic, likely-polymorphic or VUS, while 13% (9/70) were classified as pathogenic by HmtVar (Supplementary Table S1).

In short, a total of 117 oncocytic neoplasms were re-screened to reveal whether additional somatic pathogenic mutations that had gone previously undetected due to a lack of information or to the use of a single pathogenicity predictor could be retrieved. Particularly for tRNA mutations, proper assessment of a mutation's disruptive effect was difficult in the past. The advantages of using HmtVar are the 11% increase in cases bearing one or more pathogenic mtDNA mutations (90/117, 77% with an average of 1.6 mutations per patient, including frameshift and nonsense mutations), underlining how the fraction of mutated samples had been previously underestimated (77/117, 66% with an average of 1.2 mutations per patient, including frameshift and nonsense mutations), helping strengthen the association between a genetic lesion and a well-characterised phenotype.

### Interface

HmtVar is accessible at https://www.hmtvar.uniba.it/ and offers both a web interface and a RESTful API to query its content. Using the Query web page, the database can be interrogated using several search parameters, from broader criteria to narrower ones.

After performing a query, the list of variants retrieved is shown in an html table reporting variants and basic associated information; it is possible to sort this list as well as to show a given number of results on each page. Further details for each variant are available through the Variant Cards, displayed by clicking on the mutation code.

**Figure 5.**
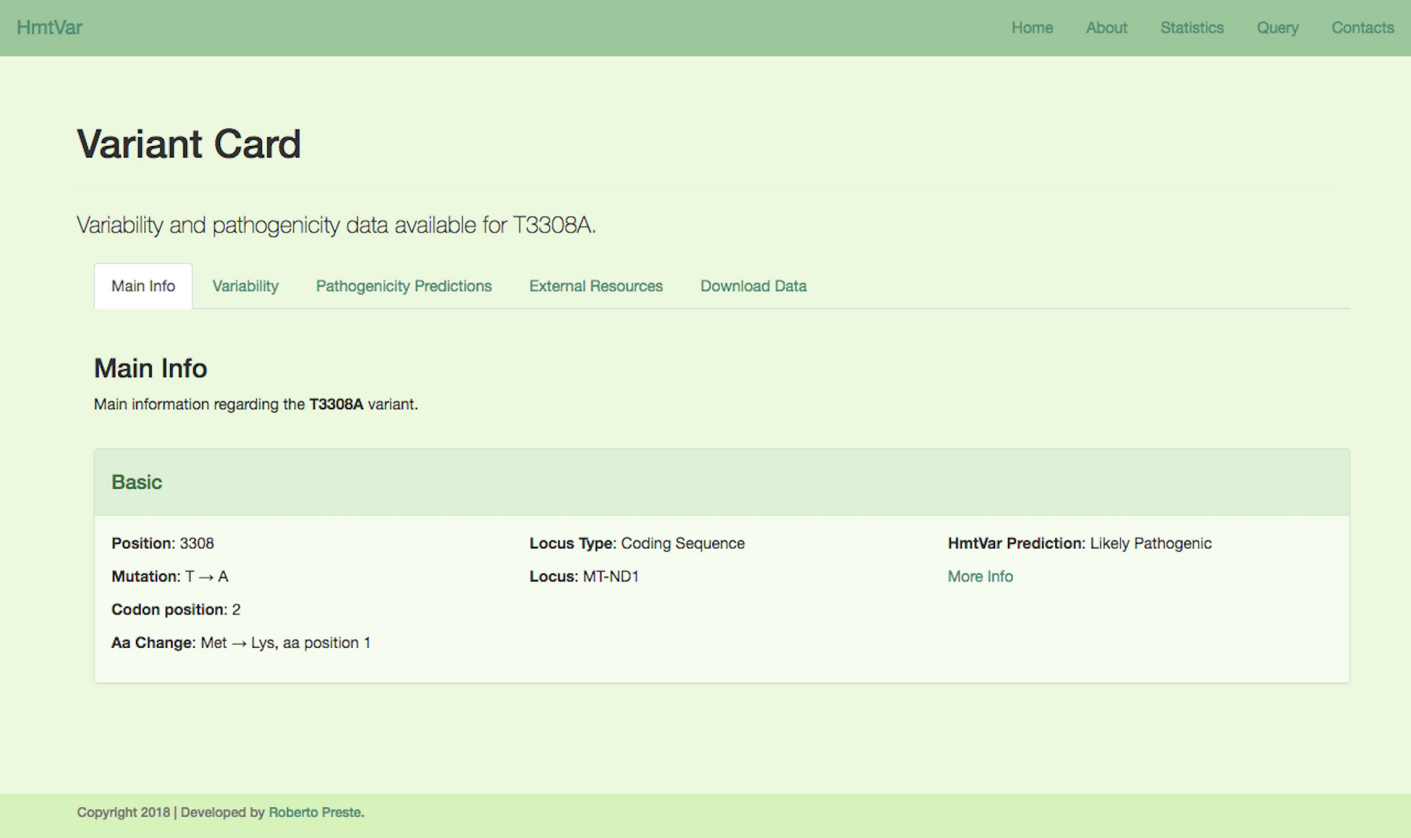
HmtVar Variant Card: Main Info Tab. It reports the variant's basic information such as its location, the consequent amino acidic change for non-synonymous variants or specific structural details for tRNA variants, haplogroups and macro-haplogroups code if the variant is associated to a specific haplogroup according to Phylotree build 17 (http://phylotree.org/index.htm), as well as HmtVar pathogenicity prediction.

Variant Cards gather all the information available for the selected variant and arrange it neatly using different tabs, such as Main Info (Figure 5), Variability (Supplementary Figure S1), Pathogenicity Predictions (Supplementary Figure S2), External Resources (Supplementary Figure S3, provided by 24,25,26,27,28,29), Download Data.

An extensive description regarding data, structure and usage of the database as well as variant statistics is presented in the About and Statistics sections (https://www.hmtvar.uniba.it/about, https://www.hmtvar.uniba.it/stats).

## API

In addition to the Query page, HmtVar allows the retrieval of variants using a dedicated Application Programming Interface (API), allowing users to access and download data in a programmatic manner. Valid API calls will return one or more results formatted as a JSON string, for easy parsing of information.

HmtVar API queries can be made to https://www.hmtvar.uniba.it/api/main/, and further information can be found on the https://www.hmtvar.uniba.it/apis page.

When returning a single variant, the API will provide the complete set of information available for the specific variant, exactly like the data shown in the Download Data tab of a Variant Card.

When returning a list of variants, on the other hand, each element in the list will report the URL to directly access the variant's complete data, as well as a limited set of basic information.

To further distribute mitochondrial variant information, HmtVar also offers a second form of API in addition to that previously described, created to be compliant with the RD-Connect platform specifications, so that it can be exploited by RD-Connect to collect and integrate HmtVar variant data into their platform. RD-Connect (30) is a comprehensive platform that integrates databases, patient registries, data analysis tools and biobanks for rare disease research. In order to collect and integrate data from a broad range of bioinformatic resources, RD-Connect has established a common API that data providers can adopt; using a set of standardised arguments for API calls, RD-Connect is then able to retrieve, parse, integrate and redistribute third-party data.

Further details can be found on https://www.hmtvar.uniba.it/apis.

### Conclusions

HmtVar offers a wide range of information regarding human mitochondrial genome variants, representing one of the most comprehensive resources for mitochondrial genomic variation studies. The broad set of mtDNA data hosted on several different online sources was exploited to build a unique aggregated database to fulfill the clinician's needs when looking for pathogenicity information on mitochondrial variants.

Pathogenicity predictions for variants located in mitochondrial tRNA and protein-coding genes allow the assessment of the harmfulness of each variation based on a pathogenicity consensus obtained using different tools and experimental information. Thanks to the classification provided by HmtVar it was possible to reveal non-synonymous and tRNA pathogenic mutations in oncocytic tumors, known to be characterized by disruptive mtDNA mutations, at a higher frequency than previously reported for the same samples.

The occurrence of pathogenic variants in healthy subjects (7%) should not be considered as due to the overestimation of pathogenicity derived from the algorithm implemented, but rather to the fact that such variants display a very low allele frequency in healthy subjects (see DST values in Table 2b). This implies a higher probability that the healthy subjects carrying the pathogenic variant may belong to haplogroups other than that in which the pathogenic-defined variant determines a pathologic phenotype. Moreover, the high number of likely polymorphic variants reflects the fact that these variants have an under threshold disease score but an allele frequency above the threshold; this is explained by the prevalence of a particular allele in specific population lineages. This information can be easily obtained from HmtVar annotations, together with the third-party data supplied to help clinicians with pathogenicity assessment.

As regards tRNA variants, it is worth mentioning that the MitoTip database provides detailed information and assignment of pathogenicity for tRNA variants. However, a close collaboration between HmtVar, Mitomap and MSeqDR curators is agreed and the exchange of information will be settled through the implementation of specific API interfaces.

Future implementations will focus on extending HmtVar's dataset beyond protein-coding and tRNA gene variants to embrace the whole set of potential variations with respect to the rCRS reference sequence. Calculations of pathogenicity prediction for rRNA and regulatory region variants will be performed, in order to offer a full overview of human mitochondrial variability and pathogenicity. Pathogenicity assignment will be further improved considering the guidelines that the MSeqDR-ClinGen mtDNA expert panel (https://goo.gl/68xaEN) is defining in agreement with the Mitomap and MSeqDR curators.

## Supplementary Material

Supplementary Data

## References

[1] Gorman G.S., Chinnery P.F., DiMauro S., Hirano M., Koga Y., McFarland R., Suomalainen A., Thorburn D.R., Zeviani M., Turnbull D.M. Mitochondrial diseases. Nat. Rev. Dis. Primers. 2016; 2:16080. 27775730 10.1038/nrdp.2016.80

[2] Hatakeyama H., Goto Y.-I. Concise review: heteroplasmic mitochondrial DNA mutations and mitochondrial diseases: toward iPSC-Based disease modeling, drug discovery, and regenerative therapeutics. Stem Cells. 2016; 34:801–808. 26850516 10.1002/stem.2292

[3] Williams M., Caino M.C. Mitochondrial dynamics in type 2 diabetes and cancer. Front. Endocrinol. (Lausanne). 2018; 9:211. 29755415 10.3389/fendo.2018.00211 PMC5934432

[4] van Oven M., Kayser M. Updated comprehensive phylogenetic tree of global human mitochondrial DNA variation. Hum. Mutat. 2009; 30:E386–E394. 18853457 10.1002/humu.20921

[5] Lott M.T., Leipzig J.N., Derbeneva O., Xie H.M., Chalkia D., Sarmady M., Procaccio V., Wallace D.C. mtDNA variation and analysis using mitomap and mitomaster. Curr. Protoc. Bioinformatics. 2013; 44:1–26. 25489354 10.1002/0471250953.bi0123s44 PMC4257604

[6] Sonney S., Leipzig J., Lott M.T., Zhang S., Procaccio V., Wallace D.C., Sondheimer N. Predicting the pathogenicity of novel variants in mitochondrial tRNA with MitoTIP. PLoS Comput. Biol. 2017; 13:e1005867. 29227991 10.1371/journal.pcbi.1005867 PMC5739504

[7] Falk M.J., Shen L., Gonzalez M., Leipzig J., Lott M.T., Stassen A.P.M., Diroma M.A., Navarro-Gomez D., Yeske P., Bai R. Mitochondrial Disease Sequence Data Resource (MSeqDR): a global grass-roots consortium to facilitate deposition, curation, annotation, and integrated analysis of genomic data for the mitochondrial disease clinical and research communities. Mol. Genet. Metab. 2015; 114:388–396. 25542617 10.1016/j.ymgme.2014.11.016 PMC4512182

[8] Clima R., Preste R., Calabrese C., Diroma M.A., Santorsola M., Scioscia G., Simone D., Shen L., Gasparre G., Attimonelli M. HmtDB 2016: data update, a better performing query system and human mitochondrial DNA haplogroup predictor. Nucleic Acids Res. 2017; 45:D698–D706. 27899581 10.1093/nar/gkw1066 PMC5210550

[9] Benson D.A., Cavanaugh M., Clark K., Karsch-Mizrachi I., Lipman D.J., Ostell J., Sayers E.W. GenBank. Nucleic Acids Res. 2013; 41:D36–D42. 23193287 10.1093/nar/gks1195 PMC3531190

[10] Andrews R.M., Kubacka I., Chinnery P.F., Lightowlers R.N., Turnbull D.M., Howell N. Reanalysis and revision of the Cambridge reference sequence for human mitochondrial DNA. Nat. Genet. 1999; 23:147. 10508508 10.1038/13779

[11] Rubino F., Piredda R., Calabrese F.M., Simone D., Lang M., Calabrese C., Petruzzella V., Tommaseo-Ponzetta M., Gasparre G., Attimonelli M. HmtDB, a genomic resource for mitochondrion-based human variability studies. Nucleic Acids Res. 2012; 40:D1150–D1159. 22139932 10.1093/nar/gkr1086 PMC3245114

[12] Attimonelli M., Accetturo M., Santamaria M., Lascaro D., Scioscia G., Pappadà G., Russo L., Zanchetta L., Tommaseo-Ponzetta M. HmtDB, a human mitochondrial genomic resource based on variability studies supporting population genetics and biomedical research. BMC Bioinformatics. 2005; 6:S4. 10.1186/1471-2105-6-S4-S4 PMC1866381 16351753

[13] Santorsola M., Calabrese C., Girolimetti G., Diroma M.A., Gasparre G., Attimonelli M. A multi-parametric workflow for the prioritization of mitochondrial DNA variants of clinical interest. Hum. Genet. 2016; 135:121–136. 26621530 10.1007/s00439-015-1615-9 PMC4698288

[14] Diroma M.A., Lubisco P., Attimonelli M. A comprehensive collection of annotations to interpret sequence variation in human mitochondrial transfer RNAs. BMC Bioinformatics. 2016; 17:338. 28185569 10.1186/s12859-016-1193-4 PMC5123245

[15] Yarham J.W., Al-Dosary M., Blakely E.L., Alston C.L., Taylor R.W., Elson J.L., McFarland R. A comparative analysis approach to determining the pathogenicity of mitochondrial tRNA mutations. Hum. Mutat. 2011; 32:1319–1325. 21882289 10.1002/humu.21575

[16] Gasparre G., Bonora E., Tallini G., Romeo G. Molecular features of thyroid oncocytic tumors. Mol. Cell. Endocrinol. 2010; 321:67–76. 20184940 10.1016/j.mce.2010.02.022

[17] Kurelac I., MacKay A., Lambros M.B.K., Di Cesare E., Cenacchi G., Ceccarelli C., Morra I., Melcarne A., Morandi L., Calabrese F.M. Somatic complex I disruptive mitochondrial DNA mutations are modifiers of tumorigenesis that correlate with low genomic instability in pituitary adenomas. Hum. Mol. Genet. 2013; 22:226–238. 23049073 10.1093/hmg/dds422

[18] Iommarini L., Calvaruso M.A., Kurelac I., Gasparre G., Porcelli A.M. Complex I impairment in mitochondrial diseases and cancer: parallel roads leading to different outcomes. Int. J. Biochem. Cell Biol. 2013; 45:47–63. 22664328 10.1016/j.biocel.2012.05.016

[19] Mayr J.A., Meierhofer D., Zimmermann F., Feichtinger R., Kögler C., Ratschek M., Schmeller N., Sperl W., Kofler B. Loss of complex I due to mitochondrial DNA mutations in renal oncocytoma. Clin. Cancer Res. 2008; 14:2270–2275. 18413815 10.1158/1078-0432.CCR-07-4131

[20] Porcelli A.M., Ghelli A., Ceccarelli C., Lang M., Cenacchi G., Capristo M., Pennisi L.F., Morra I., Ciccarelli E., Melcarne A. The genetic and metabolic signature of oncocytic transformation implicates HIF1alpha destabilization. Hum. Mol. Genet. 2010; 19:1019–1032. 20028790 10.1093/hmg/ddp566

[21] Simonnet H., Demont J., Pfeiffer K., Guenaneche L., Bouvier R., Brandt U., Schagger H., Godinot C. Mitochondrial complex I is deficient in renal oncocytomas. Carcinogenesis. 2003; 24:1461–1466. 12844484 10.1093/carcin/bgg109

[22] Lang M., Vocke C.D., Merino M.J., Schmidt L.S., Linehan W.M. Mitochondrial DNA mutations distinguish bilateral multifocal renal oncocytomas from familial Birt-Hogg-Dubé tumors. Mod. Pathol. 2015; 28:1458–1469. 26428318 10.1038/modpathol.2015.101 PMC4628590

[23] Calabrese C., Simone D., Diroma M.A., Santorsola M., Guttà C., Gasparre G., Picardi E., Pesole G., Attimonelli M. MToolBox: a highly automated pipeline for heteroplasmy annotation and prioritization analysis of human mitochondrial variants in high-throughput sequencing. Bioinformatics. 2014; 30:3115–3117. 25028726 10.1093/bioinformatics/btu483 PMC4201154

[24] Landrum M.J., Lee J.M., Benson M., Brown G., Chao C., Chitipiralla S., Gu B., Hart J., Hoffman D., Hoover J. ClinVar: public archive of interpretations of clinically relevant variants. Nucleic Acids Res. 2016; 44:D862–D868. 26582918 10.1093/nar/gkv1222 PMC4702865

[25] Amberger J.S., Bocchini C.A., Schiettecatte F., Scott A.F., Hamosh A. OMIM.org: Online Mendelian Inheritance in Man (OMIM®), an online catalog of human genes and genetic disorders. Nucleic Acids Res. 2015; 43:D789–D798. 25428349 10.1093/nar/gku1205 PMC4383985

[26] Sherry S.T., Ward M.H., Kholodov M., Baker J., Phan L., Smigielski E.M., Sirotkin K. dbSNP: the NCBI database of genetic variation. Nucleic Acids Res. 2001; 29:308–311. 11125122 10.1093/nar/29.1.308 PMC29783

[27] 1000 Genomes Project Consortium Auton A., Brooks L.D., Durbin R.M., Garrison E.P., Kang H.M., Korbel J.O., Marchini J.L., McCarthy S., McVean G.A. A global reference for human genetic variation. Nature. 2015; 526:68–74. 26432245 10.1038/nature15393 PMC4750478

[28] Sudmant P.H., Rausch T., Gardner E.J., Handsaker R.E., Abyzov A., Huddleston J., Zhang Y., Ye K., Jun G., Fritz M.H.-Y. An integrated map of structural variation in 2, 504 human genomes. Nature. 2015; 526:75–81. 26432246 10.1038/nature15394 PMC4617611

[29] Pütz J., Dupuis B., Sissler M., Florentz C. Mamit-tRNA, a database of mammalian mitochondrial tRNA primary and secondary structures. RNA. 2007; 13:1184–1190. 17585048 10.1261/rna.588407 PMC1924894

[30] Thompson R., Johnston L., Taruscio D., Monaco L., Béroud C., Gut I.G., Hansson M.G., ’t Hoen P.-B.A., Patrinos G.P., Dawkins H. RD-Connect: an integrated platform connecting databases, registries, biobanks and clinical bioinformatics for rare disease research. J. Gen. Intern. Med. 2014; 29:S780–S787. 25029978 10.1007/s11606-014-2908-8 PMC4124112

[31] Siepel A., Bejerano G., Pedersen J.S., Hinrichs A.S., Hou M., Rosenbloom K., Clawson H., Spieth J., Hillier L.W., Richards S. Evolutionarily conserved elements in vertebrate, insect, worm, and yeast genomes. Genome Res. 2005; 15:1034–1050. 16024819 10.1101/gr.3715005 PMC1182216

[32] Cooper G.M., Stone E.A., Asimenos G., Comparative Sequencing Program NISC, Green E.D., Batzoglou S., Sidow A. Distribution and intensity of constraint in mammalian genomic sequence. Genome Res. 2005; 15:901–913. 15965027 10.1101/gr.3577405 PMC1172034

