# Supplementary material for: HmtVar: a new resource for human mitochondrial variations and pathogenicity data
Source: Nucleic Acids Res. 2018 Oct 29;47(Database issue):D1202–10. doi: 10.1093/nar/gky1024 (PMC6323908; doi:10.1093/nar/gky1024)
Supplement: Supplementary Data [file gky1024_supplemental_files.zip › Suppl_Legends.pdf]

Supplementary figure 1 – HmtVar Variant Card: Variability Tab. It shows nucleotide and amino-acidic variability values, for both healthy and diseased genomes; in the same tab, healthy, diseased and continent-specific allele frequencies are also reported.

Supplementary figure 2 – HmtVar Variant Card: Pathogenicity Predictions Tab. It shows a more detailed view of pathogenicity annotated in HmtVar inclusive of the global disease score, as well as pathogenicity scores calculated by the predictors used to estimate the DS value.

Supplementary figure 3 – HmtVar Variant Card: External Resources Tab. It shows a set of additional variant information coming from online resources focused on diseases (Mitomap (5), ClinVar (24), OMIM (25)), population studies (dbSNP (26) and 1000Genomes (27, 28)) and structural information (Mamit-tRNA (29)). Links to the original sources of information are always provided for consistency.

Supplementary Table 1 – Oncocytoma identified variants.
