# Supplementary figures and images for: HmtVar: a new resource for human mitochondrial variations and pathogenicity data
Source: Nucleic Acids Res. 2018 Oct 29;47(Database issue):D1202–10. doi: 10.1093/nar/gky1024 (PMC6323908; doi:10.1093/nar/gky1024)

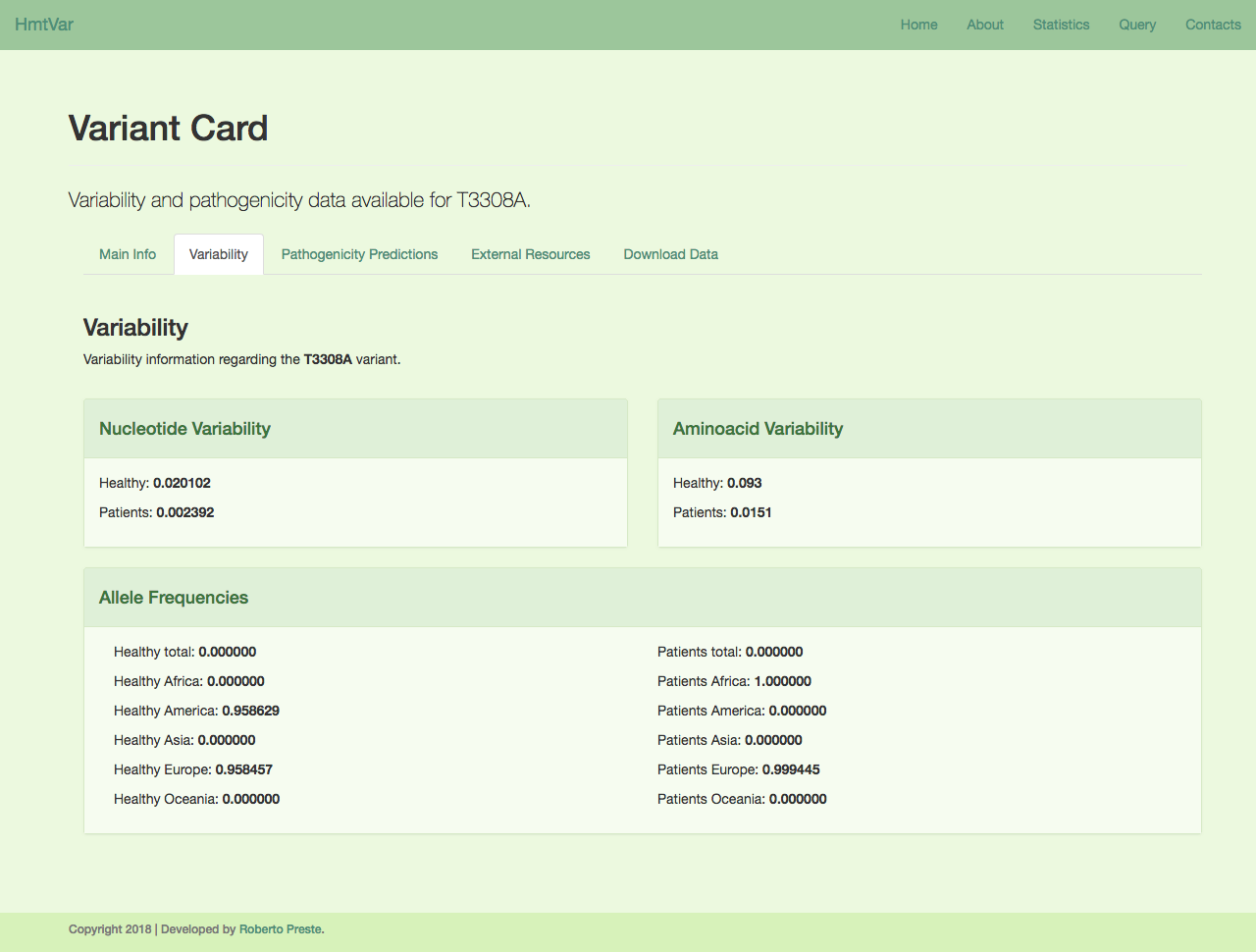

Supplement: Supplementary Data [file gky1024_supplemental_files.zip › SF1.png]

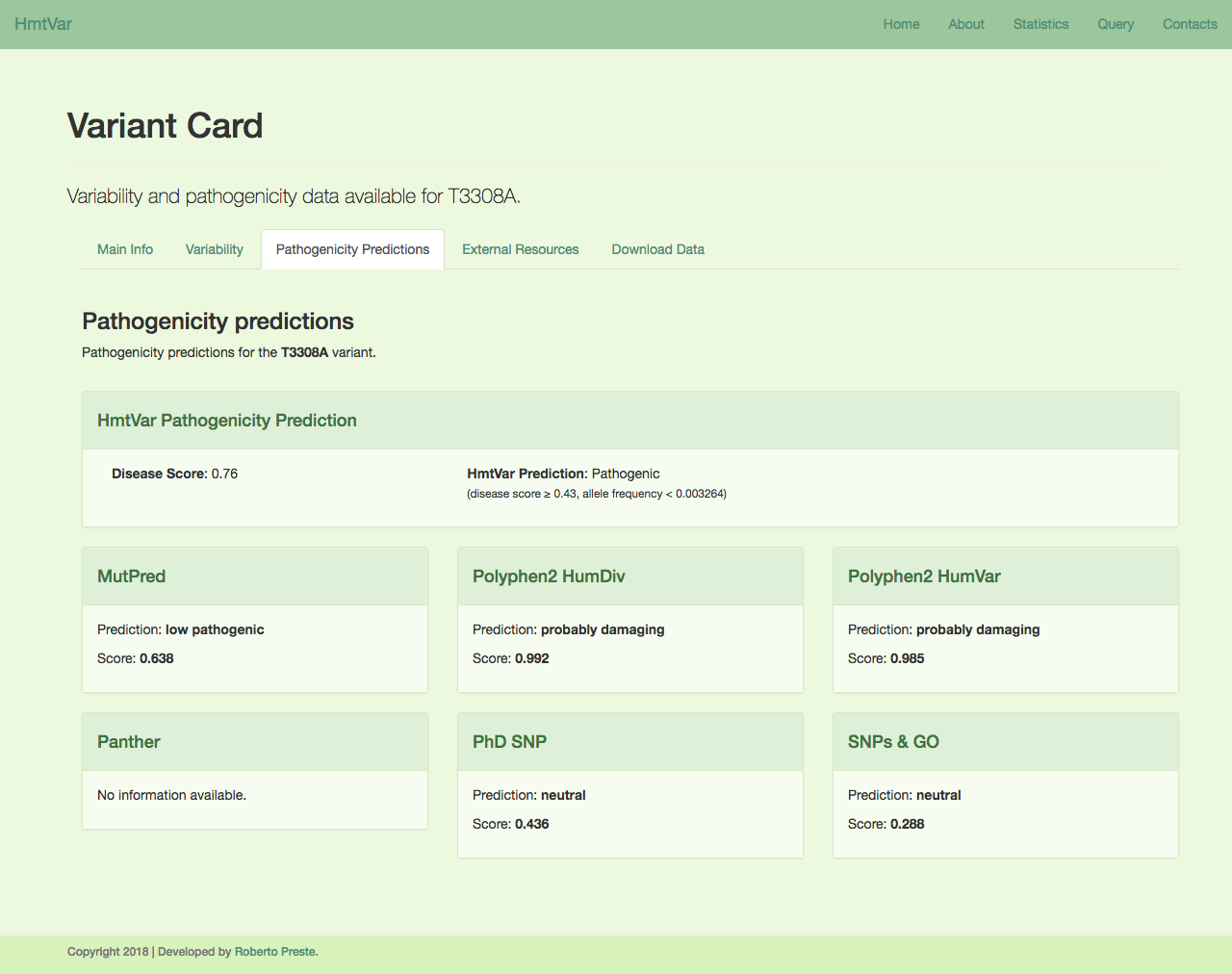

Supplement: Supplementary Data [file gky1024_supplemental_files.zip › SF2.png]

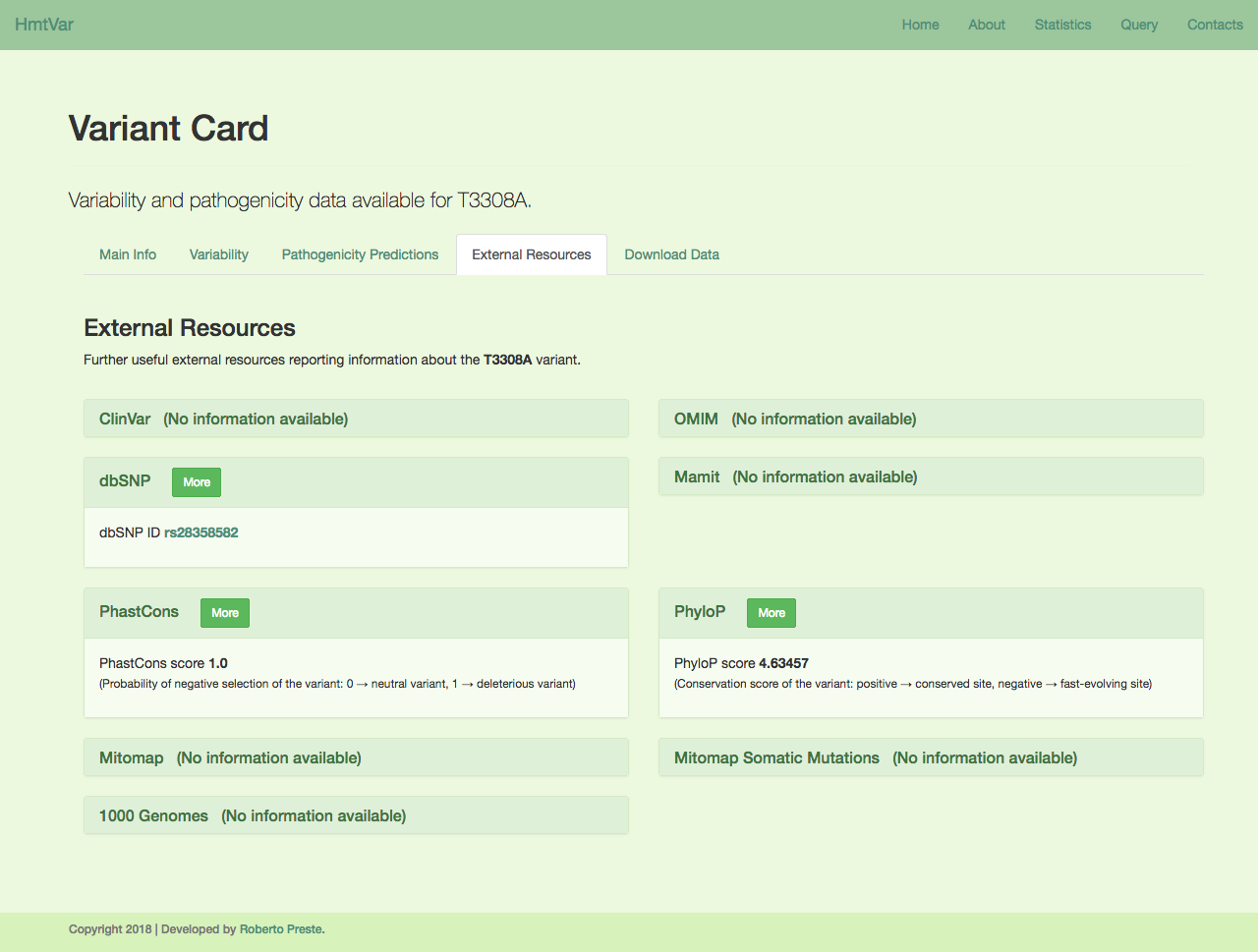

Supplement: Supplementary Data [file gky1024_supplemental_files.zip › SF3.png]
